# Conditional deficiency of m6A methyltransferase Mettl14 in substantia nigra alters dopaminergic neuron function

**DOI:** 10.1111/jcmm.16740

**Published:** 2021-07-21

**Authors:** Yan Teng, Zhihao Liu, Xingmin Chen, Yanzhuo Liu, Fan Geng, Weidong Le, Haisong Jiang, Lu Yang

**Affiliations:** ^1^ Institute of Neurology Sichuan Provincial People's Hospital University of Electronic Science and Technology of China Chengdu China; ^2^ School of Medicine University of Electronic Science and Technology of China Chengdu China

**Keywords:** dopaminergic neuron, m6A, Mettl14, tyrosine hydroxylase

## Abstract

N6‐Methyladenosine (m6A) is the most prevalent internal modification in messenger RNAs (mRNAs) of eukaryotes and plays a vital role in post‐transcriptional regulation. Recent studies demonstrated that m6A is essential for the normal function of the central nervous system (CNS), and the deregulation of m6A leads to a series of CNS diseases. However, the functional consequences of m6A deficiency within the dopaminergic neurons of adult brain are elusive. To evaluate the necessity of m6A in dopaminergic neuron functions, we conditionally deleted Mettl14, one of the most important part of m6A methyltransferase complexes, in the substantia nigra (SN) region enriched with dopaminergic neurons. By using rotarod test, pole test, open‐field test and elevated plus maze, we found that the deletion of Mettl14 in the SN region induces impaired motor function and locomotor activity. Further molecular analysis revealed that Mettl14 deletion significantly reduced the total level of m6A in the mRNA isolated from SN region. Tyrosine hydroxylase (TH), an essential enzyme for dopamine synthesis, was also down‐regulated upon Mettl14 deletion, while the activation of microglia and astrocyte was enhanced. Moreover, the expression of three essential transcription factors in the regulation of TH including Nurr1, Pitx3 and En1, with abundant m6A‐binding sites on their RNA 3’‐untranslated regions (UTR), was significantly decreased upon Mettl14 deletion in SN. Our finding first confirmed the significance of m6A in maintaining normal dopaminergic function in the SN of adult mouse.

## 1 INTRODUCTION

N6‐Methyladenosine (m6A) is one of the most abundant RNA modifications that is involved in the control of sophisticated gene expression under physiological and pathophysiological conditions. m6A of mRNA is edited by a conserved methyltransferase complex that includes Mettl3 (methyltransferase‐like 3), Mettl14 (methyltransferase‐like 14) and WTAP (Wilms tumour 1–associated protein).^1^, ^2^

Dopamine (DA), the most abundant neurotransmitter in the brain, is involved in various physiological functions of the central nervous system (CNS), such as motor functions, motivation and reward‐related learning. There are three important dopaminergic pathways in the CNS, the most important of which is the nigrostriatal pathway, which originates in the Substantia nigra pars compacta (SNpc) and projects to the caudate and putamen. This pathway mainly participates in the regulation of movement.^3^, ^4^ Recently, a growing number of studies demonstrated the important role of m6A in the development and functions of the nervous system. Abnormal m6A has been reported to be involved in the regulation of several behaviour functions, including sensorimotor function, locomotor activity and leaning.^1^, ^2^, ^5^, ^6^ Dopamine, as a major regulatory neurotransmitter of these behaviour functions, its relationship with m6A is still indistinct.

In the present study, we used lentivirus‐mediated deletion of Mettl14 in SN region, to study the necessity of m6A in maintaining the survival and relative function in dopaminergic neurons.

## 2 MATERIALS AND METHODS

### 2.1 Mice

As previously described,^7^ C57BL/6J background Mettl14‐loxp mice were generated by using the CRISPR/cas9‐based genome‐editing system. Animal procedures were approved by the Institutional Animal Care and Use Committee of School of Medicine, University of electronic science and technology of China. The details of all experimental process were provided in Appendix S1.

### 2.2 Immunofluorescence (IF)

The 40 μm coronal serial brain sections were generated using a freezing microtome (Leica Instruments). Mouse monoclonal anti‐NeuN (Abcam, ab104224) and rabbit monoclonal anti‐METTL14 (Sigma‐Aldrich, HPA038002) were used to perform immunostaining targeted to NeuN and METTL14.

### 2.3 Immunohistochemistry (IHC)

Immunohistochemistry assay was operated by using DAB detection kit (Streptavidin‐Biotin, ZSGB‐BIO, SP‐9000‐D) according to the manufacturer's manual. TH primary antibody (ProteinTech, 25859‐I‐AP) was used to detect the expression of TH in brain sections.

### 2.4 Dot blot

Anti‐m6A primary antibody (Millipore, ABE572) was used, and m6A level was detected by HRP chemiluminescence kit under chemiluminescence imaging analysis system.

### 2.5 Western blot (WB)

Anti‐METTL14 (Sigma, HPA038002), anti‐TH (ProteinTech, 25859‐I‐AP), anti‐GFAP (Novus Biologicals, NB300‐141), anti‐Iba1(Abcam, ab178847), anti‐Nurr1(Santa Cruz Biotechnology, sc‐81345), anti‐Pitx3 (Santa Cruz Biotechnology, sc‐19307X), anti‐En1(Santa Cruz Biotechnology, sc‐66876) and anti‐β‐actin (ProteinTech, 60008‐1‐Ig) antibodies were used to detect protein expression.

### 2.6 Statistical analysis

Data were expressed as mean values ± standard deviation (SD). Statistical significance was assessed using unpaired Student's *t* test. *P* < .05 was considered that there were significant differences between the groups.

## 3 RESULTS

### 3.1 Viral‐mediated deletion of Mettl14 in Substantia nigra impaired mice motor function and locomotor activity

**FIGURE 1:**
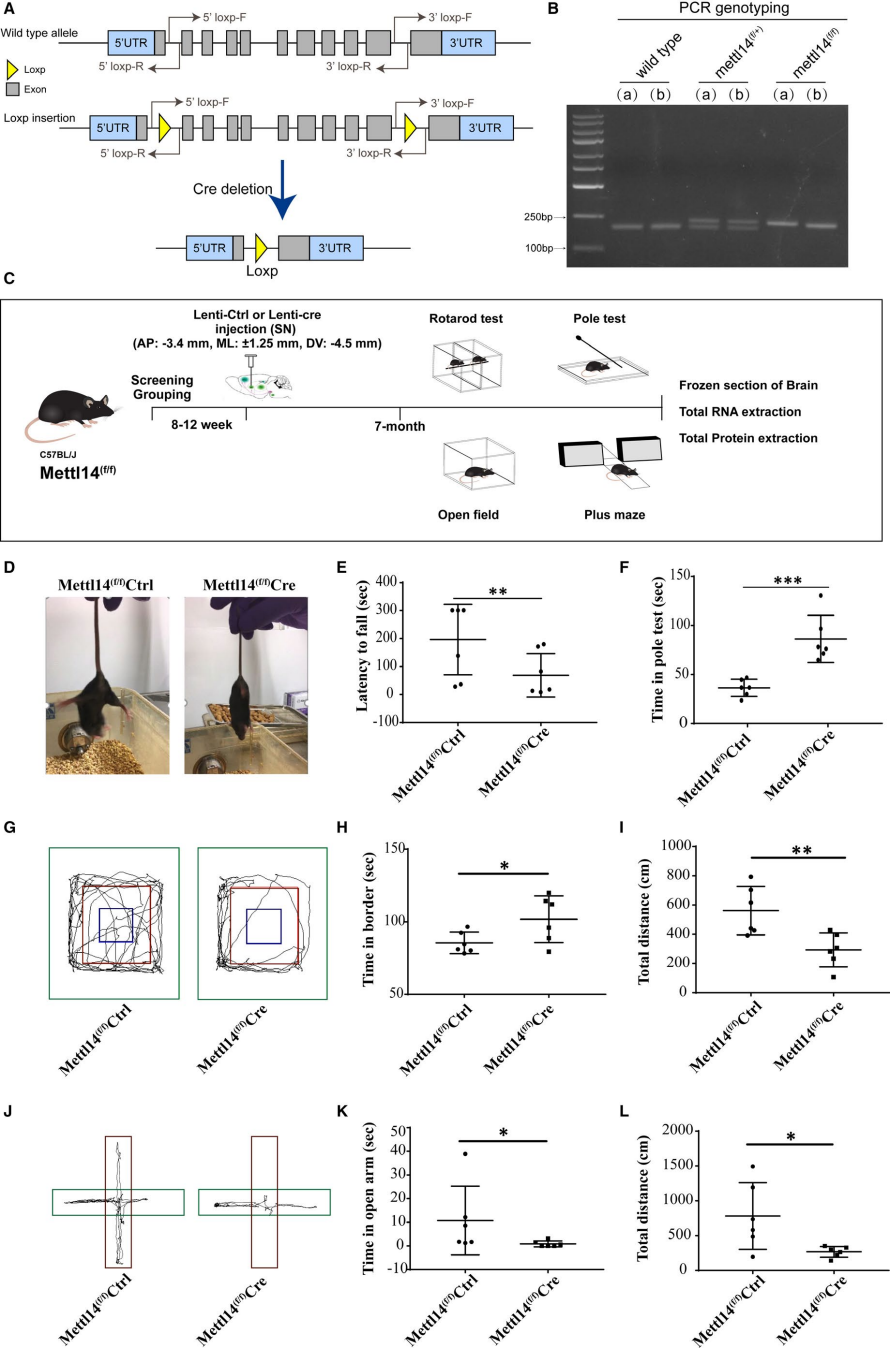
Viral‐mediated deletion of Mettl14 in Substantia nigra impaired mice motor function and locomotor activity. (A) Mettl14 deficiency was made by using loxp‐cre system, and the region from second exon to tenth exon would be deleted if Cre recombinase is existed. (B) Example of genotyping result. The PCR products by using 5’loxp‐F and 5’loxp‐R primers were separated by agar gel electrophoresis, and a 230 bp band will be detected if loxp sequence was inserted while 198 bp will be observed in wild type. We marked homozygote of the floxed Mettl14 gene as Mettl14^(f/f)^ and heterozygote as Mettl14^(f/+)^. (C) Experimental plan of the present study. One group of mice (Mettl14^(f/f)^Cre) was injected with lentivirus packaging the Cre recombinase (Lenti‐Cre) into SN region (AP: −3.4 mm, ML: ±1.25 mm, DV: −4.5 mm) to induce SN conditional Mettl14 deletion. The control group (Mettl14^(f/f)^Ctrl) received lentivirus without Cre recombinase (Lenti‐Ctrl). Five months later, the behavioural functions of mice were examined by rotarod test, pole test, open‐field test and elevated plus maze. Subsequently, mice brains were harvested. (D) Visual observation of mice. (E) A computer‐controlled rotarod apparatus with a rod (7 cm diameter) was set to accelerate from 0 to 40 revolutions per minute (rpm) for 30 sec and sustain 40 rpm for 5 min, and the time to fall was recorded (latency to fall). (F) Mice were placed on the bottom or top of the pole (40 cm length, at a 45‐degree angle to the ground), the time used from bottom to top (t1) or from top to bottom (t2) was recorded, and the sum of t1 and t2 was calculated as time in pole test. (G) An open‐field chamber with tetrahedral enclosing walls (total zone: diameter of 40 × 40 cm) was divided into three parts: border (green), periphery (red) and centre (blue). The time in border (H) and total distance in open‐field chamber zone (I) were calculated. (J) Mice were placed on the centre of elevated plus maze with an open arm (red, 40cm length) and a closed arm (green, 40cm length). Time in open arm (K) and total distance in total plus maze zone (L) were calculated. * *P* < .05, ** *P* < .01 and *** *P* < .001 vs Mettl14^(f/f)^Ctrl (n = 6)

To explore the effect of m6A in dopaminergic neuron function, we generated SN conditional Mettl14 knockout mice by using loxp‐cre system through in vivo stereotactic injection of lentivirus (Figure 1A,B. Coordinates: AP: −3.4 mm, ML: ±1.25 mm, DV: −4.5 mm). The experimental plan of the present study was shown in Figure 1C. We found a marked decrease in motor function and locomotor activity in conditional Mettl14 knockout mice. The Mettl14^(f/f)^Cre mice group exhibited a marked decline in grasping ability while lifting mice tail (Figure 1D). Both the rotarod (Figure 1E and Video S1) and pole test (Figure 1F and Videos [Link], [Link]) demonstrated that the motor function of Mettl14^(f/f)^Cre mice was significantly impaired compared with Mettl14^(f/f)^Ctrl mice. The locomotor activity was tested by open‐field test (Figure 1G) and elevated plus maze (Figure 1J). The open‐field test showed that Mettl14^(f/f)^Cre mice spend more time in border of open‐field chamber (Figure 1H), and they moved shorter distance in zone than Mettl14^(f/f)^Ctrl mice (Figure 1I). The elevated plus maze results showed that Mettl14^(f/f)^Cre mice took less time in open arm (Figure 1K), and they moved shorter distance in zone than Mettl14^(f/f)^Ctrl mice (Figure 1L).

### 3.2 Viral‐mediated deletion of Mettl14 in SN reduced m6A level

**FIGURE 2:**
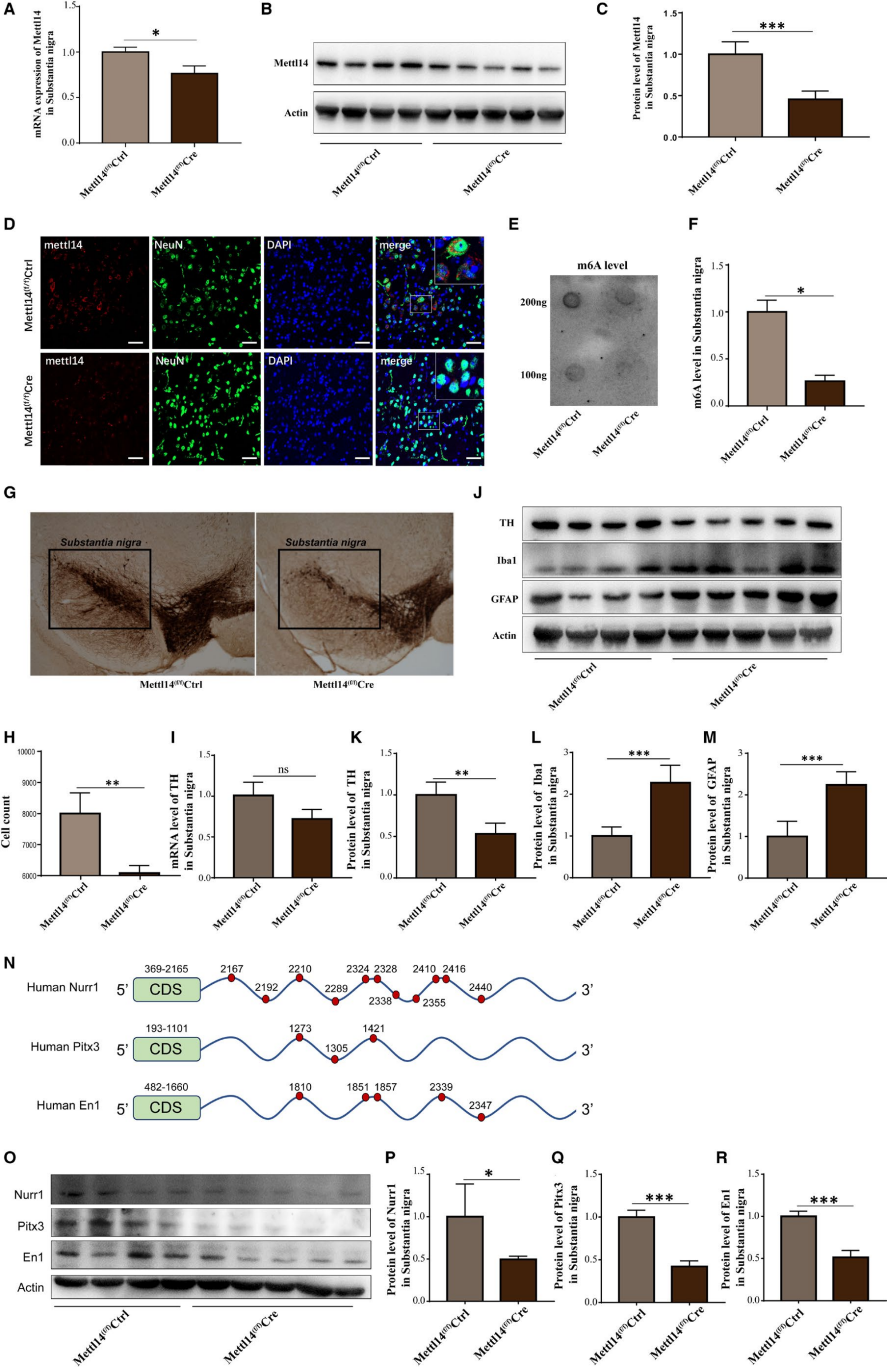
Viral‐mediated deletion of Mettl14 in SN reduced TH expression, enhanced the activation of microglia and astrocytes, and decreased the expression of Nurr1, Pitx3 and En1. (A)The RNA expression of Mettl14 in SN was detected by RT‐qPCR. (B and C) The protein level of METTL14 was ascertained by Western blot. (D) IF assay showed the METTL14 expression in neurons of SN. Images were captured at 20× (red: Mettl14, green: NeuN, blue: DAPI, bar=40μm). (E and F) m6A level of total RNA from SN was determined by dot blot assay. (G) IHC assay was utilized to stain tyrosine hydroxylase (TH) in SN. (H) Quantification of TH‐positive cell number from (G) by cell count. The RNA expression (I) and the protein level (J and K) of TH were ascertained by RT‐qPCR and Western blot. (J, L and M) The protein levels of Iba1 and GFAP were ascertained by Western blot. (N) Schematic diagram of m6A‐binding targets on mRNA of Nurr1, Pitx3 and En1. The green box represents the coding sequence (CDS), and the blue line represents the 3’ untranslated region (3’UTR) of mRNA. The red dot and related number indicate the position of m6A‐binding sites. (O‐R) The protein levels of Nurr1, Pitx3 and En1 were measured by Western blot. “ns” represents that there is no statistical significance, * *P* < .05, ** *P* < .01 and *** *P* < .001 vs Mettl14^(f/f)^Ctrl. (n = 4 for Mettl14^(f/f)^Ctrl and n = 5 for Mettl14^(f/f)^Cre)

Real‐time quantitative PCR (RT‐qPCR) (Figure 2A) and Western blot (Figure 2B,C) showed the expression of Mettl14 was significantly reduced in SN region of Mettl14^(f/f)^Cre mice. By immunofluorescence assay, we further validated an obvious decrease in METTL14 level in SN neurons of Mettl14^(f/f)^Cre mice by staining METTL14 with neuronal nuclei (NeuN), which has been widely used as a marker for post‐mitotic neurons (Figure 2D). Meanwhile, dot blot assay displayed a markedly decrease in m6A level in SN region of Mettl14^(f/f)^Cre mice (Figure 2E,F). These results illustrated that Mettl14 expression and m6A modification were effectively reduced through in vivo stereotactic injection of lentivirus.

### 3.3 Viral‐mediated deletion of Mettl14 in SN reduced TH expression and enhanced the activation of microglia and astrocytes

Tyrosine hydroxylase (TH) is a monooxygenase that acts as a rate‐limiting enzyme in the formation of L‐dopamine, loss of function or reduced expression of TH directly affects the synthesis and secretion of dopamine.^4^ We found an obvious reduction in TH expression in SN of Mettl14^(f/f)^Cre mice compared with Mettl14^(f/f)^Ctrl mice by both IHC (Figure 2G,H) and WB (Figure 2J,K) assays. RT‐qPCR result shows a downward trend in TH in Mettl14^(f/f)^Cre mice compared with Mettl14^(f/f)^Ctrl mice (Figure 2I). These data suggest that mettl14 is implicated in dopamine synthesis. Meanwhile, we also detected significant activation of microglia and astrocytes, as the expression of ionized calcium‐binding adapter molecule 1 (Iba1) and glial fibrillary acidic protein (GFAP) was obviously increased in Mettl14^(f/f)^Cre mice compared with Mettl14^(f/f)^Ctrl mice (Figure 2J,L,M).

### 3.4 Viral‐mediated deletion of Mettl14 reduced the expression of Nurr1, Pitx3 and En1 in SN

Nuclear Receptor‐Related Protein 1 (Nurr1), pituitary homeobox 3 (Pitx3) and engrailed1 (En1) are three widely known transcription factors that are essential for the TH expression and related dopaminergic functions.^8^, ^9^ Interestingly, we noticed abundant m6A‐binding sites on the mRNA 3’‐untranslated regions (UTR) of Nurr1, Pitx3 and En1 from a sequence‐based m6A modification site predictor SRAMP website (http://www.cuilab.cn/sramp/), especially Nurr1 (Figure 2N). Therefore, we detected the expression of Nurr1, Pitx3 and En1 by WB and found that protein expression of Nurr1, Pitx3 and En1 was significantly reduced in the SN of Mettl14^(f/f)^Cre mice compared with Mettl14^(f/f)^Ctrl mice (Figure 2O‐R). This suggested that these transcription factors may be the essential molecules regulated upon m6A modification to impact TH expression and dopaminergic functions.

## 4 DISCUSSION

N6‐Methyladenosine is identified as the most abundant chemical modification on mammalian mRNA to date and plays an important role in brain function. Mettl14, as an essential methyltransferase of m6A, has been demonstrated to play an indispensable role for the neurogenesis during development.^10^, ^11^, ^12^, ^13^ Besides its roles in neurogenesis, Mettl14 is reported to be involved in the function of striatal neurons in the adult brain.^14^ In the present study, we first reported the role of m6A in dopaminergic neurons and related function. Our results demonstrated that Mettl14 deletion in SN region alters dopaminergic functions that may be related to reduced TH expression. Additionally, another in vitro study demonstrated that m6A reduction could cause dopaminergic neuron apoptosis through elevating oxidative stress and Ca^2+^ influx.^15^ These results suggest that m6A modification plays a vital role to support the normal function of dopaminergic neuron via maintaining the expression of TH in dopaminergic neurons. Moreover, three important transcription factors Nurr1, Pitx3 and En1 might be the target of Mettl14‐mediated m6A modification to affect the expression of TH and its relative functions.

Our findings provide the first evidence to support the essential role of Mettl14‐mediated m6A modification in dopaminergic neurons; further studies are needed to explore the underlying molecular mechanisms of m6A‐regulated neuronal functions.

## CONFLICT OF INTEREST

The authors confirm that there are no conflicts of interest.

## AUTHOR CONTRIBUTIONS

**Yan Teng:** Data curation (lead); Methodology (equal); Writing‐original draft (equal). **Zhihao Liu:** Data curation (lead). **Xingmin Chen:** Data curation (supporting). **Yanzhuo Liu:** Data curation (supporting). **Fan Geng:** Data curation (supporting). **Weidong Le:** Conceptualization (supporting). **Haisong Jiang:** Conceptualization (equal); Resources (equal). **Lu Yang:** Conceptualization (equal); Data curation (supporting); Methodology (equal); Resources (equal); Writing‐original draft (lead).

## Supporting information

App S1

Video S1

Video S2

Video S3

## Data Availability

The data that support the findings of this study are available from the corresponding author upon reasonable request.
