# Supplementary material for: Conditional deficiency of m6A methyltransferase Mettl14 in substantia nigra alters dopaminergic neuron function
Source: J Cell Mol Med. 2021 Jul 21;25(17):8567–72. doi: 10.1111/jcmm.16740 (PMC8419180; doi:10.1111/jcmm.16740)
Supplement: Supplementary file 1 — App S1 [file JCMM-25-8567-s003.docx]

**Appendix S1**

**Supplementary information for**

**Conditional deficiency of m6A methyltransferase Mettl14 in** **Substantia nigra alters dopaminergic neuron function**

Yan Teng^1,2^*, Zhihao Liu^1,2^*, Xingmin Chen^2^, Yanzhuo Liu^2^, Fan Geng^2^, Weidong Le^1,2^, Haisong Jiang^1,2#^, Lu Yang^1,2#^

^1^Institute of Neurology, Sichuan Provincial People's Hospital, University of Electronic Science and Technology of China, Chengdu, Sichuan 610072, China

^2^School of medicine, University of Electronic Science and Technology of China, Chengdu, Sichuan, 610054, China

* Those two authors contributed equally to this work.

# Those two authors are corresponding authors.

Correspondence: Dr. Lu Yang or Dr. Haisong Jiang, School of Medicine, Institute of Neurology, Sichuan Provincial People's Hospital, University of Electronic Science and Technology of China, Chengdu, China, E-mail: [lyang@uestc.edu.cn](mailto:lyang@uestc.edu.cn) & jhsarchangle@hotmail.com

**Materials and Methods**

**Mice**

As previously described (H. B. Li et al., 2017), C57BL/J background mettl14-loxp mice were generated by flanking the mettl14 gene (the first and the last introns) with two loxp sequences using the CRISPR/cas9 based genome-editing system. The new born mice were genotyped by using two pairs of primers (5’loxp-F and 5’loxp-R) or (3’loxp-F and 3’loxp-R). All mice were littermates and co-housed for any experiments described. Animal procedures were approved by the Institutional Animal Care and Use Committee of School of Medicine, University of electronic science and technology of China.

**Viral Injection**

8-12 weeks old mice were anesthetized by using 2% isofluorane, Mice were injected with a lenti-virus (10^8^ TU/mL, GenePharma Co. Shanghai, China) into Substantia nigra region under sterile conditions (AP: -3.4 mm, ML: ±1.25 mm, DV: -4.5 mm), 2μL of lenti-virus with EGFP-Cre-recombinase or EGFP Ctrl was delivered at a rate of 0.5μL /min. The lenti-virus has been centrifuged at high speed (>10000 g) overnight before injection. All of the mice were alive after injection.

**Rotarod test**

A computer-controlled rotarod apparatus (SA102, SANS, China) with a rod (seven cm diameter) was set to accelerate from 0 to 40 revolutions per minute (rpm) for 30 sec and sustained 40 rpm for five minutes, and the time to fall was recorded (latency to fall).

**Pole test**

Mice was placed on the bottom or top of the pole (40cm length, at a 45-degree angle to the ground), the time used from bottom to top (t1) or from top to bottom (t2) was recorded, the sum of t1 and t2 was calculated as time in pole test.

**Open Field**

An open field chambers with tetrahedral enclosing walls (diameter of 40 × 40 cm) was divided into three parts: border, periphery and center. The motion track of mice was recorded by infrared beams under a computer connected camera. Data were collected for two min per mouse. The time in border and total distance in open field chamber were calculated as locomotor activity.

**Elevated-plus maze.**

Mice were placed on the center of plus maze with an open arm (40cm-length) and a closed arm (with walls, 40cm-length). The motion track of mice was recorded the same as open field. Data were collected for two min per mouse. Time in open arm and total distance in plus maze were calculated as locomotor activity.

**Immunofluorescence (IF)**

Brain tissues of mice were fixed with 4% formaldehyde and transferred to a 30% sucrose for 24 hours twice, 40 μm coronal serial brain sections were made using a freezing microtome (CM1860, Leica Instruments). Mouse monoclonal anti-NeuN (1:200, Abcam, Cat# ab104224) and rabbit monoclonal anti-mettl14 (1:200, Sigma-Aldrich, Cat# HPA038002) were incubated with tissue sections for 72 hours, followed by incubation of goat anti-mouse Alexa Fluor 488 (A-11029, Thermo Fisher) and goat anti-rabbit Alexa Fluor 594 (A-11012, Thermo Fisher) conjugated secondary antibodies at 1:500 for one hour at room temperature. The cells were marked using nuclear counterstaining with DAPI (Sigma).

**Immunohistochemistry (IHC)**

Brain tissue sections were obtained as IF assay. IHC assay was operated by using DAB detection kit (Streptavidin-Biotin) (ZSGB-BIO, Cat# SP-9000-D) according manufacturer's manual. Tissue sections were treated with H_2_O_2_ for ten minutes to eliminate endogenous peroxidase. After washing with PBS, sections were then blocked with 5% normal goat serum in PBS that contain 0.3% Triton X-100 (blocker) for 30 minutes at room temperature. TH primary antibody (1:300, Protein tech, Cat#25859-I-AP) diluted in blocker was added and incubated at 4℃ overnight. The next day, antibody was removed by washing with PBS three time, followed by incubation with biotin and Streptavidin Peroxidase labeled secondary antibodies for 20 minutes at room temperature respectively. Finally, DAB solution was used for staining.

**RT-qPCR**

Mice brains were dissected, and the SN regions were separated, Total RNA of SN was extracted by using Trizol reagent (Invitrogen) according manufacturer's manual. cDNA for qPCR was synthesized by using HiScript III RT SuperMix kit (Vazyme, Cat#R323-01). ChamQ Universal SYBR qPCR Master Mix (Vazyme, Cat#Q711-02) was used for gene amplification and the mRNA expression was meansured by detecting Syer green signal. Primers used in this study are shown as following, GAPDH: (Forward,5’-CTA CAC TGA GGA CCA GGT TGT C-3’) and (Reverse,5’-GTT ATT ATG GGG GTC TGG GAT GG-3’), Mettl14: (Forward, 5’-CTG AGA GTG CGG ATA GCA TTG-3’) and (Reverse, 5’-GAG CAG ATG TAT CAT AGG AAG CC-3’), TH: (Forward, 5’- GAC AGT CCT CAC ACC ATC CG-3’, Reverse: 5’- CTG TGG GTG GTA CCC TAT GC-3’).

**Dot-blot**

Total RNA from SN region was obtained the same as RT-qPCR. 100ng and 200ng RNA in 2μL volume was dropped on a NT membrane. Membrane was then crosslinked by UV exposure (Ultraviolet Crosslinker, Analytikjena), followed by blocking with 5% non-fat milk (BD Biosciences, Cat#232100) diluted in TBST for one hour at room temperature. Anti-m6A primary antibody (1:1000, Millipore, Cat# ABE572) was added and incubated with NT membrane at 4℃ overnight. The next day, m6A antibody was removed by washing with TBST and membrane was incubated with secondary antibody for one hours at room temperature. The protein expression was detected by HRP chemiluminescence kit (Millipore, WBKLS0100) under chemiluminescence imaging analysis system (Tanon, Tanon-5200). Quantification of the blots was assessed by Image J.

**Western blot**

Mice brains were dissected, and the SN regions were separated. Total protein of SN was extracted by using RIPA solution. Protein concentration were measured by a BCA kit (Solarbio, Cat# CA1210). Loading buffer was added in protein samples and boiled at 98℃ for five minutes. The equal amount of protein samples was electrophoresed in a sodium dodecyl sulfate-polyacrylamide gel (10%-12.5%) followed by transferring to PVDF membranes (Millipore). PVDF membranes were blocked with 5% non-fat milk (BD Biosciences, Cat#232100) or 5% BSA (Amresco, Cat# 0332) for one hour at room temperature. And then the blots were probed with anti-mettl14 (1:1000, Sigma, Cat# HPA038002), anti-TH (1:300, Protein tech, Cat#25859-I-AP), anti-GFAP (Novus Biologicals, NB300-141), anti-Iba1(Abcam, ab178847), anti-Nurr1(Santa Cruz Biotechnology, sc-81345), anti-Pitx3 (Santa Cruz Biotechnology, sc-19307X), anti-En1(Santa Cruz Biotechnology, sc-66876) and anti-β-actin (1:5000, Proteintech, cat# 60008-1-Ig) primary antibodies and secondary antibodies diluted with 1×TBST. Finally, the blots were detected the same as dot blot.

**Statistical analysis**

Data were expressed as mean values ± standard deviation (SD). All data analysis was tested by unpaired student’s t-test. p< 0.05 was considered that there were significant differences between the groups.
